# Assessment of changes in intra-abdominal pressure and cardiac output induced by liver compression in healthy anesthetized spontaneously breathing dogs

**DOI:** 10.1371/journal.pone.0315491

**Published:** 2024-12-12

**Authors:** Daeyun Seo, Seongsoo Lim, Beomkwan Namgoong, Heesung Uhm, Hyeajeong Hong, Nanju Lee, Isong Kim, Seunghun Heo, Ji Hwan Kang, Cheyoun Kim, Hayoung Shin, Jiwoong Her, Min Su Kim

**Affiliations:** 1 Department of Veterinary Clinical Sciences, College of Veterinary Medicine and Research institute for Veterinary Science, Seoul National University, Seoul, South Korea; 2 Department of Veterinary Clinical Sciences, College of Veterinary Medicine, The Ohio State University, Columbus, Ohio, United States of America; Universidade de Trás-os-Montes e Alto Douro: Universidade de Tras-os-Montes e Alto Douro, PORTUGAL

## Abstract

**Objective:**

Liver compression (LC) has been proposed to predict fluid responsiveness in human pediatric patients. Because the evaluation of fluid responsiveness through LC depends on the mechanism of increased intra-abdominal pressure (IAP), understanding the impact of LC on IAP, cardiac output (CO), and respiratory parameters is essential. Thus, this study aimed to investigate the effects of LC on these parameters.

**Methods:**

The present study used six healthy beagles. All dogs were anesthetized with isoflurane and allowed to breathe spontaneously in dorsal recumbency. After instrumentation, LC was performed at four different pressures in a sequential, non-randomized manner: (1) 10 mmHg, approximately half of the minimum value within the range; (2) 22 mmHg, a commonly used pressure within the range; (3) 44 mmHg, twice the pressure of the minimum value within the range; and (4) 60 mmHg, twice the pressure of the maximum value within the range. At each pressure, CO via transthoracic echocardiography, IAP, and cardiorespiratory parameters were measured before, during, and after LC.

**Results:**

Overall, our results showed that the IAP was significantly increased at all pressures during LC (P < 0.001), yielding a linear correlation between LC pressure and IAP (P < 0.001; r² = 0.89). The maximum IAP during LC was 7 mmHg, and intra-abdominal hypertension was not induced. LC at 22 mmHg significantly increased the IAP by 1.7 mmHg, but did not significantly alter the CO or respiratory parameters.

**Conclusions:**

This is the first study to evaluate the effects of LC on IAP, CO, and respiratory parameters in healthy, anesthetized, and spontaneously breathing dogs. Our findings indicate that applying LC with a commonly used pressure may have a low risk of inducing intra-abdominal hypertension and related complications. Further studies are required to explore the use of LC in various clinical settings.

## Introduction

The abdominal cavity is a relatively compliant space; however, owing to its confined nature, an increase in the intracavitary volume or a decrease in the extracavitary compliance can lead to elevated intra-abdominal pressure (IAP). Factors such as increased abdominal wall tone caused by pain, anxiety, abdominal masses, obesity, mechanical ventilation, or external pressure applied to the abdomen can directly impair abdominal wall compliance, resulting in increased IAP [1].

Liver compression (LC) has been proposed in human pediatric patients to predict fluid responsiveness (FR). LC utilizes external pressure applied to the mid-abdomen to increase IAP, which temporarily redistributes blood from the intra-abdominal organs and vessels to the central circulation, thereby increasing the preload and cardiac output (CO). In human pediatric studies, a LC of 22–30 mmHg successfully predicted FR [2–4]. A recent experimental study demonstrated that LC significantly increased stroke volume (SV) and CO in healthy, anesthetized dogs with hypovolemia, and could be used as a novel method to evaluate FR [5].

However, an excessive increase in IAP can lead to intra-abdominal hypertension (IAH), which can displace the diaphragm cranially and compress the heart and caudal vena cava, thereby reducing venous return and CO. IAH is defined as a marked increase in IAP above 12 mmHg, and severe IAH is a significant and well-recognized cause of morbidity and mortality in both human and veterinary medicine [1]. In one study conducted on normotensive pigs, an increase in IAP of 7.5–15 mmHg resulted in a temporary increase in CO; however, an increase in IAP to 30 mmHg decreased CO [6]. In addition, IAH can impair ventilation by reducing diaphragmatic excursion, decreasing functional residual and total lung capacities, and increasing work of breathing [1]. A study in healthy dogs revealed that an IAP below 12 mmHg had no significant effect on respiratory parameters [7], but IAP above 20 mmHg reduced tidal volume (VT) and induced respiratory acidosis [8]. Severe and acute IAH can directly lead to the failure of both the cardiac (arrhythmias and myocardial infarction) and pulmonary systems (ventilatory impairment), resulting in sudden death. Therefore, the monitoring of IAP in critically ill patients is important in both human and veterinary medicine [1, 9, 10].

As the evaluation of FR through LC relies on the mechanism of increased IAP, it is essential to understand the effects of LC on IAP, CO, and respiratory parameters; however, research on the impact of LC on these parameters in both humans and animals remains limited. Thus, the present study aimed to (1) evaluate the effects of LC at various pressures on IAP and CO, and (2) assess the effects of LC on respiratory parameters. Additionally, we investigated the effects of LC on hemodynamic parameters. We hypothesized that (1) as LC pressure increases, IAP would increase proportionally, and (2) the LC pressure used in clinical practice would not induce IAH, and significantly affect CO and respiratory parameters in healthy, anesthetized, and spontaneously breathing dogs.

## Materials and methods

### Animals

All procedures, animal care and handling were approved by the Ethics Committee for Experimental Animals of Seoul National University (SNU-230725-2). All dogs were housed individually in stainless-steel cages equipped with feeders, drinkers, and feces and urine collectors, in a controlled animal room maintained at 18–26°C, with a relative humidity of 30–70% and a 12-hour light-dark cycle. The animal room and cages were cleaned daily. Water was available ad libitum, and a dry commercial diet was provided twice a day to meet their daily maintenance energy requirements. Six beagles aged 17–18 months, including three intact males and three intact females, were included in this study. Each dog was confirmed to be healthy through a physical examination, complete blood count, serum chemistry panel, and thoracic and abdominal radiographic examinations. After the study, the dogs were returned to their respective cages for use in subsequent non-invasive studies.

### Anesthesia and instrumentation

Prior to the induction of anesthesia, all dogs were fasted for 12 h with free access to water. A 24-gauge IV catheter (Introcan Certo; BRAUN., Germany) was aseptically inserted into the cephalic vein with minimal physical restraint. The dogs were preoxygenated with a facemask for 5 min, and anesthesia was induced with alfaxalone (2 mg/kg, IV; Alfaxan Multidose; JUROX., Australia). Following endotracheal tube placement, the dog was positioned in dorsal recumbency, and anesthesia was maintained with isoflurane (Ifran; Hana Pharm Co. Ltd., Korea) in 100% oxygen (2 L/min). The end-tidal concentration of isoflurane (ET_Iso_), end-tidal partial pressure of carbon dioxide (PETCO_2_), and respiratory rate (RR) were monitored using an infrared gas analyzer included in a multiparameter monitor (CARESCAPE B650; GE healthcare., Finland). The ET_Iso_ was adjusted to between 1.6–1.8% to maintain spontaneous breathing. Intermittent positive pressure ventilation was performed when PETCO_2_ exceeded 60 mmHg. VT, heart rate (HR), esophageal temperature (T_eso_), and peripheral oxygen saturation of hemoglobin as measured by pulse oximetry (SpO_2_) were monitored using the same monitor. Minute ventilation (MV) was calculated by multiplying VT by RR. T_eso_ was maintained between 37.0–38.0 ℃ using a warm air blanket (Bair-Hugger; 3M United States., USA).

A 24-gauge IV catheter was aseptically placed in the dorsal pedal artery to measure the invasive blood pressure and collect blood samples. A 5-Fr double-lumen catheter (MILA international Inc., USA) was aseptically inserted into the right jugular vein to measure central venous pressure (CVP). Both catheters were connected to a pressure transducer, and arterial blood pressure and mean CVP were monitored using a multiparameter monitor (CARESCAPE B650; GE healthcare., Finland). Pressure transducers were placed at the estimated level of the right atrium, and zeroed to the atmospheric pressure. The catheters were flushed periodically with heparinized saline, and maintenance fluids were not administered because of their potential impact on hemodynamic data during the anesthetic procedure.

A 6-Fr Foley urinary catheter (YUSHIN MEDICAL Co. Ltd., Korea) was aseptically inserted into the bladder to measure the IAP using a standardized transvesical technique [11]. The urine was fully emptied and the bladder was instilled with 0.9% sodium chloride (1 mL/kg). The pressure transducer was connected to the urinary catheter using a 3-way stopcock, and the IAP was continuously monitored using a multiparameter monitor in the dorsal recumbent position. The pressure was zeroed at the mid-axillary line of the iliac crest, and the IAP was recorded at the end-expiratory phase. During LC, an increase in IAP ≥ 12 mmHg was defined as IAH.

### Liver compression technique

Based on both human and veterinary literature [2–5], the mid-abdomen of dogs was compressed for 1 min in the dorsal recumbent position using a modified compressor connected to a digital force gauge (AMF-50; Aliyiqi Co., China). In human pediatric medicine, LC is performed within the range of 22–30 mmHg. Thus, the LC pressure was determined according to the following criteria: (1) 10 mmHg, approximately half of the minimum value within the range; (2) 22 mmHg, commonly used pressure within the range; (3) 44 mmHg, twice the pressure of the minimum value within the range; and (4) 60 mmHg, twice the pressure of the maximum value within the range.

### Transthoracic echocardiographic measurement of cardiac output

**Fig 1:**
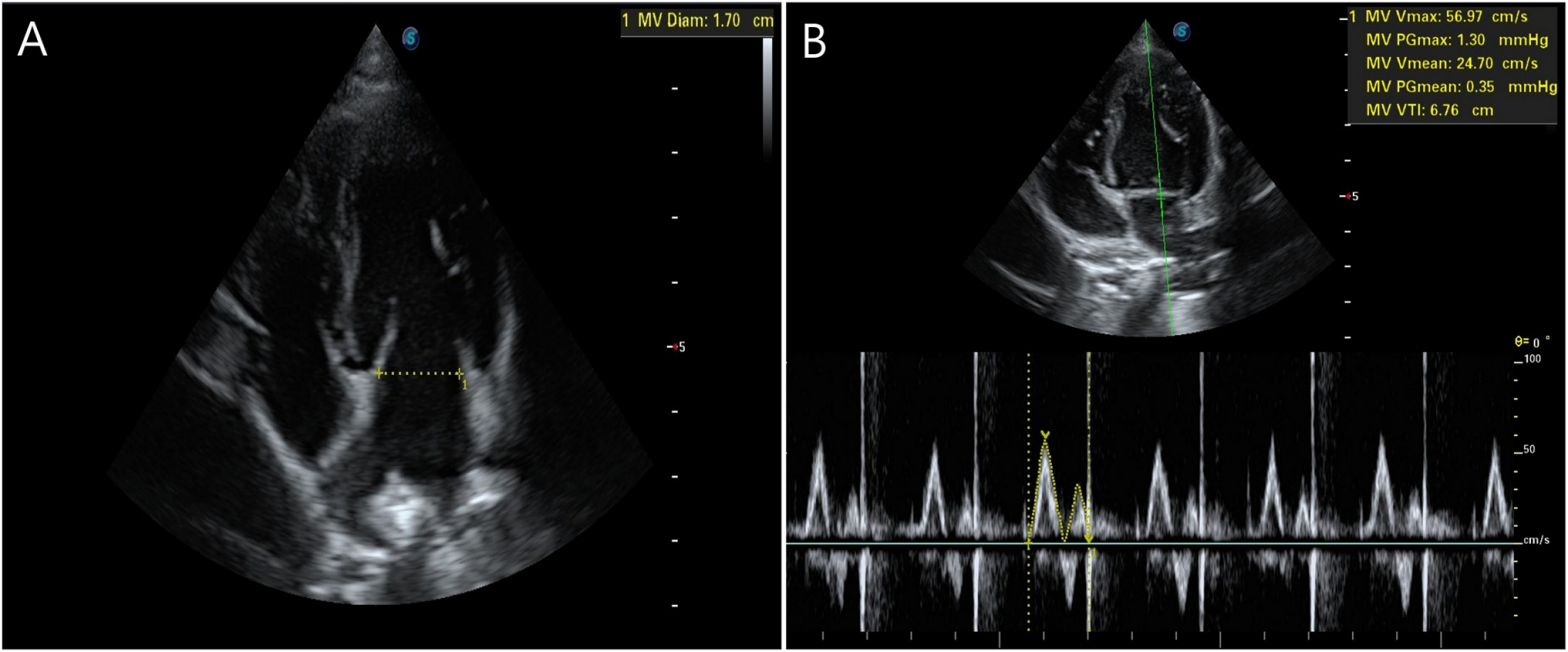
Transthoracic echocardiographic measurements of mitral valve diameter and velocity time integral in left apical four-chamber view of healthy, anesthetized, and spontaneously breathing dogs.

Transthoracic echocardiography (TTE) was performed by a single operator to minimize interobserver variation, using an ultrasound system (P20 pro; SonoScape., China), equipped with a 2–9 MHz phased array probe with dogs in the dorsal recumbency position. The mitral valve diameter was measured between the inner edges of the posterior and anterior leaflets during early to mid-diastole at mitral valve opening in the left apical four-chamber view (Fig 1), and the cross-sectional area (CSA) of the mitral valve was calculated as follows: π × (mitral valve diameter^2^/4). The mitral blood flow waveform was obtained using a pulse-wave Doppler at the level of the mitral valve, while sampling gate was placed proximal to the mitral valve leaflets.

Due to the nature of the interventions (LC), it was not feasible to blind the operator performing the TTE. To minimize potential bias, the stored images were randomized and analyzed by a single operator after all procedures were completed. The mitral valve velocity time integral (VTI) was measured by manually tracing the Doppler flow signal using electronic calipers in the ultrasound machine software (Fig 1). To minimize the hemodynamic influence of heart-lung interactions, mitral valve VTI was measured by averaging five consecutive waves over one respiratory cycle. Stroke volume (SV) was calculated as VTI × CSA, and CO was obtained by multiplying SV by HR. Systemic vascular resistance (SVR) was calculated as (MAP-CVP)/CO × 79.9 [12].

### Experimental design

**Fig 2:**
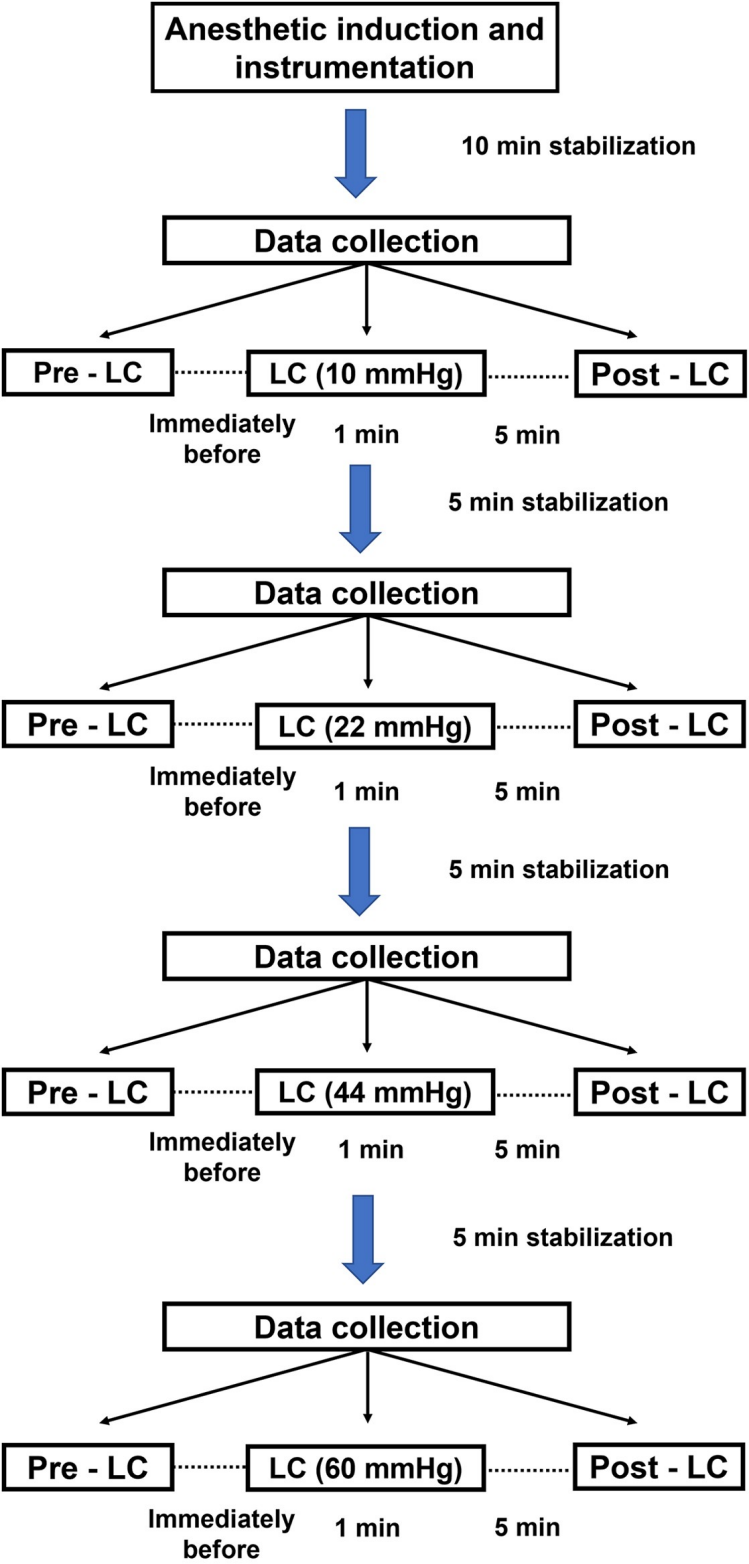
Timeline of the study design and data collection for evaluating the effects of liver compression (LC) on intra-abdominal pressure (IAP), cardiac output (CO), and respiratory parameters in healthy, anesthetized, and spontaneously breathing dogs. Following anesthetic induction and instrumentation, the dogs were allowed 10 min to stabilize cardiorespiratory variables. Data were collected at three time points: immediately before LC (pre-LC); during the application of pressure to the mid-abdomen for 1 min (LC); and 5 min after the release of pressure (post-LC). A 5-min stabilization period was provided between changes in LC pressure.

After all instrumentation was completed, 10 min was allocated to stabilize the cardiorespiratory variables. LC was performed at various pressures in a non-randomized sequence as follows: 10, 22, 44, and 60 mmHg. Between changes in LC pressure, a 5-min period elapsed prior to data collection to stabilize hemodynamic status. Each LC procedure was divided into three time points: pre-LC, immediately before LC; LC, application of pressure to the mid-abdomen for 1 min; and post-LC, 5 min after pressure release (Fig 2). At each time point, IAP, HR, MAP, mean CVP, T_eso_, SpO_2_, RR, PETCO_2_, and VT were measured, followed by VTI. All cardiorespiratory variables, except for VTI, were recorded at end-expiration. Simultaneously, arterial blood samples were collected and analyzed using a blood gas analyzer (GEM Premier 5000; Werfen., Spain).

### Recovery from anesthesia

After obtaining the final data, the arterial and jugular catheters were removed, and meloxicam (0.2 mg/kg, SC; Metacam; Labiana Life Sciences., Spain) was administered as rescue analgesia. The isoflurane vaporizer was then turned off and recovery was initiated. After extubation, the dogs were transferred to their respective kennels. The hemodynamic variables and catheter sites were monitored periodically for 48 h.

### Statistical analysis

All analyses were performed using a commercially available software (SPSS Statistics 26; IBM Corporation., USA and GraphPad Prism 8; GraphPad Software., USA). The normality of the cardiorespiratory variables was assessed by the Shapiro-Wilk test. For normally distributed variables, means and SD were calculated. Repeated-measures one-way analysis of variance (ANOVA) was used to compare differences across variables among the three time points within each pressure and among the four LC pressures. Greenhouse and Geisser corrections were applied to analyze cases in which a lack of sphericity was observed. Tukey’s post-hoc adjustment was used when multiple comparisons were performed. During LC, the correlations among IAP, LC pressure, and CO were assessed using linear regression analysis. P-values < 0.05 were considered statistically significant.

## Results

**Fig 3:**
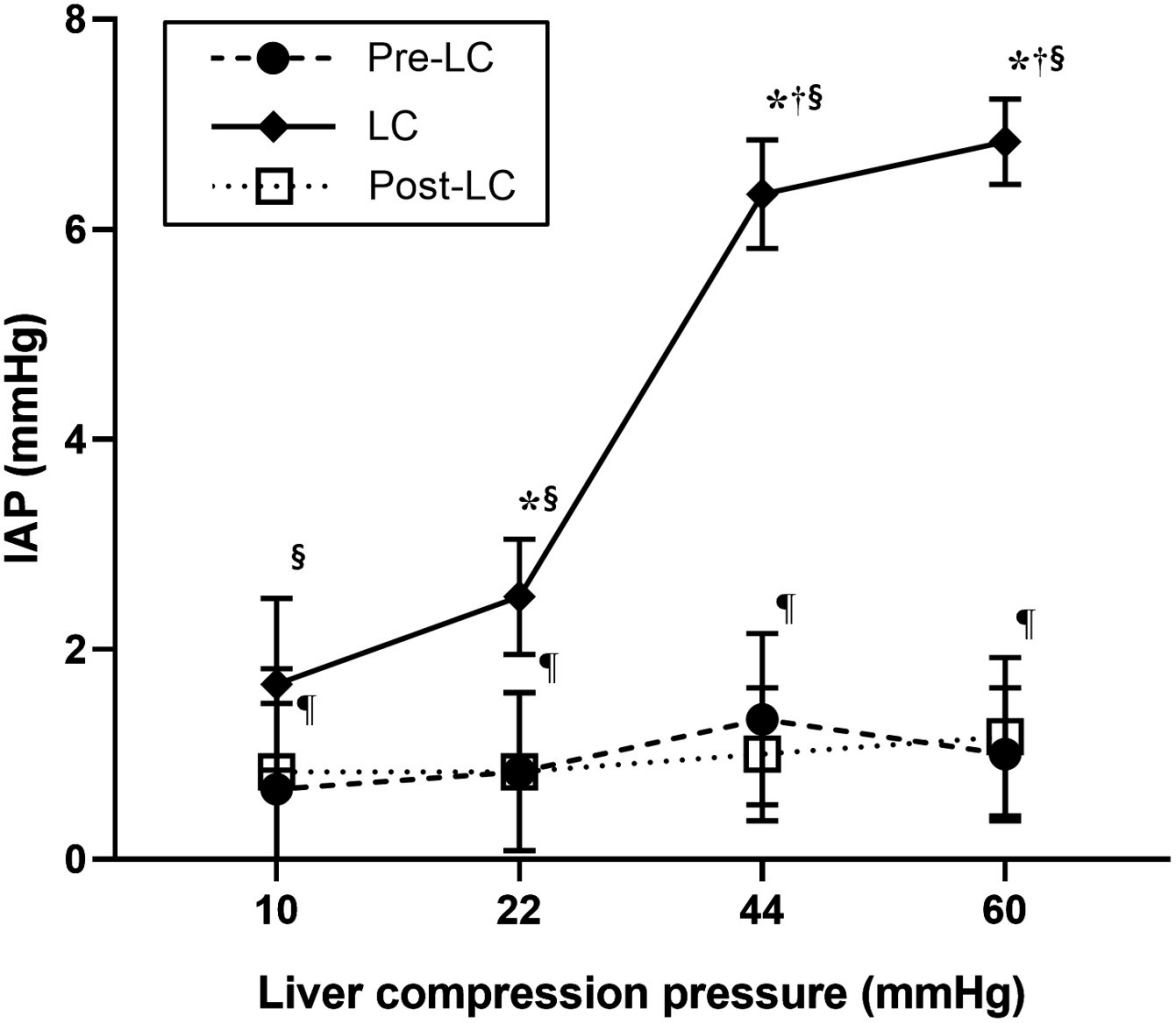
Effect of various liver compression (LC) pressures on intra-abdominal pressure (IAP) at the three time points (pre-LC, immediately before LC; LC, during the application of pressure to the mid-abdomen for 1 min; post-LC, 5 min after the release of pressure) in healthy, anesthetized, and spontaneously breathing dogs. *Significant difference from 10 mmHg at the same time point (P < 0.05). ^**†**^Significant difference from 22 mmHg at the same time point (P < 0.05). ^**§**^Significant difference from pre-LC within the same stage (P < 0.05). ^**¶**^Significant difference from LC within the same stage (P < 0.05).

**Fig 4:**
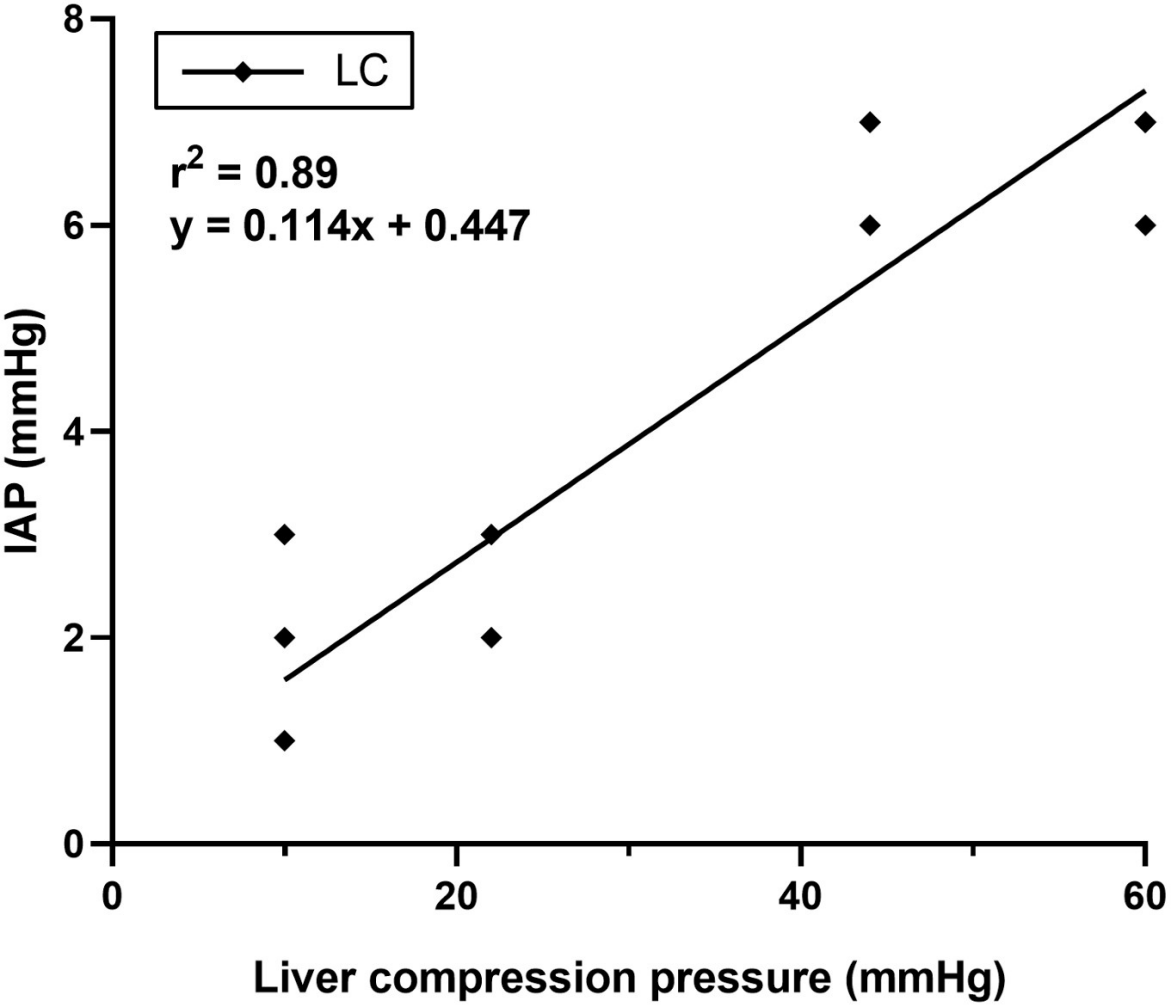
Correlation between liver compression (LC) pressures ranging from 10–60 mmHg and intra-abdominal pressure (IAP) in healthy, anesthetized, and spontaneously breathing dogs. *Significant difference from 10 mmHg at the same time point (P < 0.05). ^**†**^Significant difference from 22 mmHg at the same time point (P < 0.05). ^**§**^Significant difference from pre-LC within the same stage (P < 0.05). ^**¶**^Significant difference from LC within the same stage (P < 0.05).

All procedures were completed without complications, and all dogs recovered successfully from the anesthesia. The mean body weight was 10.3 ± 1.6 kg, and the mean mitral valve diameter was 1.72 ± 0.05 cm. During the experiments, T_eso_ was maintained between 37.1–37.7 ℃, SpO_2_ between 96–98%, and since PETCO_2_ was maintained between 38–53 mmHg, intermittent positive pressure ventilation was not performed. In all dogs, the IAP at pre-LC was 0–3 mmHg. The anesthesia time from induction to extubation was 165 ± 8 min. IAP significantly increased (P < 0.001) at all pressures (10, 22, 44, and 60 mmHg) during LC, and significantly decreased (P < 0.001) post-LC (Table 1; Fig 3). During LC, the mean IAP increased by 1, 1.7, 5, and 5.8 mmHg at 10, 22, 44, and 60 mmHg, respectively, and a linear correlation between LC pressure and IAP was observed (P < 0.001; r² = 0.89; Fig 4). Although the IAP significantly increased with higher LC pressures, no significant difference was found between 44 and 60 mmHg. At 60 mmHg LC, the maximum IAP was 7 mmHg, and no IAH was induced at any LC pressure during the experiment.

**Table 1:** Intra-abdominal pressure (IAP), cardiac output (CO), and hemodynamic variables collected in healthy, anesthetized, and spontaneously breathing dogs at various liver compression (LC) pressures. Data were collected at three time points: immediately before LC (pre-LC); during the application of pressure to the mid-abdomen for 1 min (LC); and 5 min after the release of pressure (post-LC). Data are presented as mean ± SD.

Variable	Liver compression pressure	Time of data collection		
		Pre-LC	LC	Post-LC
IAP (mmHg)	10 mmHg	0.7 ± 0.8	1.7 ± 0.8^§^	0.8 ± 1.0^¶^
	22 mmHg	0.8 ± 0.8	2.5 ± 0.6*^§^	0.8 ± 0.8^¶^
	44 mmHg	1.3 ± 0.8	6.3 ± 0.5*^†^^§^	1.0 ± 0.6^¶^
	60 mmHg	1.0 ± 0.6	6.8 ± 0.4*^†^^§^	1.2 ± 0.8 ^¶^
VTI (cm)	10 mmHg	7.32 ± 0.74	7.15 ± 0.76	7.04 ± 0.65^§^
	22 mmHg	7.08 ± 0.74	7.11 ± 0.80	7.14 ± 0.75
	44 mmHg	7.14 ± 0.59	7.34 ± 0.67	7.22 ± 0.58
	60 mmHg	7.33 ± 0.53	7.74 ± 0.66*^†^^‡^^§^	7.23 ± 0.53^¶^
CO (L/min)	10 mmHg	2.2 ± 0.5	2.2 ± 0.5	2.1 ± 0.5
	22 mmHg	2.2 ± 0.5	2.2 ± 0.5	2.2 ± 0.5
	44 mmHg	2.2 ± 0.3	2.2 ± 0.3	2.2 ± 0.4
	60 mmHg	2.3 ± 0.4	2.4 ± 0.4*^†^^§^	2.2 ± 0.3^¶^
SV (mL/beat)	10 mmHg	17.0 ± 2.4	17.0 ± 2.4	16.3 ± 2.0^§^
	22 mmHg	16.4 ± 2.2	16.4 ± 2.3	16.5 ± 2.3
	44 mmHg	16.5 ± 2.0	17.0 ± 1.9	16.7 ± 2.0
	60 mmHg	17.0 ± 1.9	17.9 ± 2.2*^†^^‡^^§^	17 ± 2.0^¶^
HR (beats/min)	10 mmHg	129 ± 20	130 ± 19	131 ± 20
	22 mmHg	134 ± 20	132 ± 19	132 ± 19
	44 mmHg	133 ± 15	132 ± 14	133 ± 17
	60 mmHg	134 ± 18	135 ± 17	133 ± 16
MAP (mmHg)	10 mmHg	80 ± 16	81 ± 15	80 ± 14
	22 mmHg	83 ± 15	84 ± 15	82 ± 13
	44 mmHg	84 ± 7	88 ± 6^§^	85 ± 9
	60 mmHg	88 ± 4	96 ± 4*^§^	90 ± 9
mean CVP (mmHg)	10 mmHg	1 ± 1	2 ± 1	1 ± 1
	22 mmHg	1 ± 1	2 ± 1^§^	1 ± 1^¶^
	44 mmHg	1 ± 1	2 ± 1^§^	1 ± 1^¶^
	60 mmHg	2 ± 1	2 ± 1	1 ± 1
SVR (dynes/s/cm^5^)	10 mmHg	2872 ± 384	2974 ± 393^§^	2966 ± 383^§^
	22 mmHg	3015 ± 424	3046 ± 419	3002 ± 339
	44 mmHg	3068 ± 346	3091 ± 367	3074 ± 376
	60 mmHg	3091 ± 401	3135 ± 394	3223 ± 396*^†^

*Significant difference from 10 mmHg at the same time point (P < 0.05).

^**†**^Significant difference from 22 mmHg at the same time point (P < 0.05).

^**‡**^Significant difference from 44 mmHg at the same time point (P < 0.05).

^**§**^Significant difference from pre-LC within the same stage (P < 0.05).

^**¶**^Significant difference from LC within the same stage (P < 0.05).

CO = Cardiac output. CVP = Central venous pressure. HR = Heart rate. IAP = Intra-abdominal pressure. MAP = Mean arterial pressure. SV = Stroke volume. SVR = Systemic vascular resistance. VTI = Velocity time integral.

The VTI, SV, and CO significantly increased by 5.6%, 5.6%, and 6.6%, respectively, at 60 mmHg LC (P < 0.001, P = 0.007, and P = 0.007, respectively) and significantly decreased post-LC (P < 0.001; Table 1). Other LC pressures did not significantly change the VTI, SV, and CO, and no linear correlation was observed between the IAP and VTI, SV, and CO.

The HR did not significantly change with LC at any pressure (Table 1). MAP increased significantly by 4 mmHg during 44 mmHg LC (P = 0.037) and by 8 mmHg during 60 mmHg LC (P = 0.030), and decreased post-LC, although this decrease was not statistically significant. The mean CVP significantly increased by 1 mmHg during LC at 22 and 44 mmHg (P = 0.002 and P < 0.001, respectively). Except for the 10 mmHg LC (P = 0.007), the SVR did not significantly change.

The VT decreased during LC at all pressures; however, this decrease was not statistically significant (Table 2). Conversely, the RR increased during LC at all pressures, with statistical significance observed only at 60 mmHg (P = 0.002). The MV increased during LC at all pressures, but the increase was not statistically significant. At 60 mmHg LC, the pH significantly increased, while PaO_2_ and PaCO_2_ significantly decreased (P = 0.007, P = 0.022, P = 0.024, respectively; Table 3). Since the fraction of inspiratory oxygen concentration (FiO_2_) was 1, the ratio of arterial partial pressure of oxygen to the fraction of inspiratory oxygen concentration (PaO_2_/FiO_2_) was the same as the PaO_2_ value. At post-LC, pH significantly decreased, and PaCO_2_ significantly increased (P = 0.002 and P = 0.018, respectively); however, PaO_2_ did not significantly change. LC did not significantly affect bicarbonate and lactate levels.

**Table 2:** Respiratory variables collected in healthy, anesthetized, and spontaneously breathing dogs at various liver compression (LC) pressures. Data were collected at three time points: immediately before LC (pre-LC); during the application of pressure to the mid-abdomen for 1 min (LC); and 5 min after the release of pressure (post-LC). Data are presented as mean ± SD.

Variable	Liver compression pressure	Time of data collection		
		Pre-LC	LC	Post-LC
VT (mL/breath)	10 mmHg	101 ± 14	97 ± 14	101 ± 13
	22 mmHg	99 ± 12	98 ± 14	99 ± 13
	44 mmHg	101 ± 12	98 ± 19	98 ± 12
	60 mmHg	101 ± 14	95 ± 15	100 ± 17
RR (breath/min)	10 mmHg	22 ± 9	24 ± 9	23 ± 8
	22 mmHg	23 ± 8	24 ± 8	23 ± 8
	44 mmHg	24 ± 10	27 ± 13	24 ± 9
	60 mmHg	24 ± 9	29 ± 12*^†^^§^	26 ± 11^¶^
MV (L/min)	10 mmHg	2.15 ± 0.59	2.21 ± 0.62	2.26 ± 0.65
	22 mmHg	2.15 ± 0.56	2.25 ± 0.62	2.23 ± 0.54
	44 mmHg	2.33 ± 0.71	2.44 ± 0.74	2.29 ± 0.67
	60 mmHg	2.27 ± 0.71	2.67 ± 0.91	2.44 ± 0.77

*Significant difference from 10 mmHg at the same time point (P < 0.05).

^**†**^Significant difference from 22 mmHg at the same time point (P < 0.05).

^**§**^Significant difference from pre-LC within the same stage (P < 0.05).

^**¶**^Significant difference from LC within the same stage (P < 0.05).

MV = Minute ventilation. RR = Respiratory rate. VT = Tidal volume.

**Table 3:** Arterial blood gas analysis in healthy, anesthetized, and spontaneously breathing dogs at various liver compression (LC) pressures. Data were collected at three time points: immediately before LC (pre-LC); during the application of pressure to the mid-abdomen for 1 min (LC); and 5 min after the release of pressure (post-LC). Data are presented as mean ± SD.

Variable	Liver compression pressure	Time of data collection		
		Pre-LC	LC	Post-LC
pH	10 mmHg	7.30 ± 0.02	7.29 ± 0.03	7.28 ± 0.02^§^
	22 mmHg	7.29 ± 0.03*	7.29 ± 0.03	7.28 ± 0.03^¶^
	44 mmHg	7.28 ± 0.02*	7.29 ± 0.04	7.28 ± 0.03
	60 mmHg	7.28 ± 0.03*	7.30 ± 0.02^§^	7.28 ± 0.03^¶^
PaO_2_/FiO_2_	10 mmHg	506 ± 23	481 ± 28	482 ± 14
	22 mmHg	480 ± 14	482 ± 37	487 ± 13
	44 mmHg	487 ± 29	473 ± 36	476 ± 35
	60 mmHg	495 ± 18	475 ± 21^§^	488 ± 25
PaO_2_ (mmHg)	10 mmHg	506 ± 23	481 ± 28	482 ± 14
	22 mmHg	480 ± 14	482 ± 37	487 ± 13
	44 mmHg	487 ± 29	473 ± 36	476 ± 35
	60 mmHg	495 ± 18	475 ± 21^§^	488 ± 25
PaCO_2_ (mmHg)	10 mmHg	48 ± 4	47 ± 4	47 ± 3
	22 mmHg	48 ± 3	46 ± 4	48 ± 3
	44 mmHg	48 ± 4	46 ± 5	47 ± 4
	60 mmHg	49 ± 3	46 ± 3^§^	49 ± 5^¶^
HCO_3_^-^ (mmol/L)	10 mmHg	23.1 ± 1.7	22.4 ± 1.2	22.3 ± 1.3
	22 mmHg	22.4 ± 1.0	21.8 ± 2.1	22.5 ± 1.5
	44 mmHg	22.3 ± 1.3	22.0 ± 1.4	22.1 ± 1.2
	60 mmHg	22.8 ± 1.2	22.2 ± 1.3	22.4 ± 1.6
Lactate (mmol/L)	10 mmHg	0.8 ± 0.4	0.8 ± 0.4	0.7 ± 0.4
	22 mmHg	0.8 ± 0.4	0.7 ± 0.4	0.8 ± 0.3
	44 mmHg	0.7 ± 0.3	0.8 ± 0.3	0.8 ± 0.2
	60 mmHg	0.7 ± 0.2	0.8 ± 0.2	0.8 ± 0.3

*Significant difference from 10 mmHg at the same time point (P < 0.05).

^**§**^Significant difference from pre-LC within the same stage (P < 0.05).

^**¶**^Significant difference from LC within the same stage (P < 0.05).

HCO_3_^-^ = Bicarbonate. PaO_2_ = Arterial partial pressure of oxygen. PaCO_2_ = Arterial partial pressure of carbon dioxide. PaO_2_/FiO_2_ = Ratio of arterial partial pressure of oxygen to the fraction of inspiratory oxygen concentration.

## Discussion

This study evaluated the effects of LC on the IAP, CO, and respiratory parameters in healthy, anesthetized, and spontaneously breathing dogs. We found that LC significantly increased IAP at all pressures; however, it did not induce IAH. During 22 mmHg LC, the IAP significantly increased by 1.7 mmHg; however, no significant changes were observed in the CO and respiratory parameters. Conversely, an IAP above 6.8 mmHg induced by 60 mmHg LC can affect CO and respiratory parameters.

LC has been proposed to predict FR in human pediatric patients by inducing transient and reversible auto-transfusion, similar to passive leg raising, which increases venous return and subsequently preload and CO [2–4]. LC could increase IAP, potentially inducing a fluid shift from the splanchnic vascular bed to the central circulation [6, 13]. However, previous studies did not measure IAP, the impact of LC on IAP has not been demonstrated. LC pressures are typically applied within the range of 22–30 mmHg; however, pressures as high as 45–50 mmHg have also been used [2–5, 14]. Therefore, in this study, LC was performed at pressures ranging from 10–60 mmHg.

A significant correlation was observed between LC pressures ranging from 10–60 mmHg and IAP; however, no significant difference in IAP was detected between 44 and 60 mmHg. This suggests that the impact on the IAP may decrease gradually beyond a LC pressure of 60 mmHg. While LC did not induce IAH in this study, the interpretation of the findings should consider the following factors: (1) this study was conducted in healthy, spontaneously breathing dogs under anesthesia, potentially yielding different outcomes in actual clinical scenarios. Increased intra-abdominal content, peritonitis, mechanical ventilation, shock, and obesity are known risk factors that decrease abdominal compliance and induce IAH [1]. Additionally, in conscious patients, LC may induce agitation and pain, which can increase abdominal muscle tone and lead to IAH [13]; (2) IAP was measured in dorsal recumbency and can vary with different body positions. In human studies, measuring the IAP in a completely supine position is recommended [15]. However, because dorsal recumbency (equivalent to the supine position in humans) is not the natural position for hospitalized dogs, lateral recumbency is preferred for measuring IAP [1, 16, 17]. In healthy dogs, IAP in lateral recumbency ranges from 0 to 3.7 mmHg. In this study, IAP before LC ranged from 0 to 3 mmHg, which is consistent with previous studies despite differences in posture [18, 19].

The LC at 10, 22, and 44 mmHg did not significantly affect VTI, SV, and CO, and no linear correlation was observed between IAP and VTI, SV, and CO. These results align with the finding that LC at 22 mmHg did not significantly change SV and CO in normovolemic dogs, indicating that under a normovolemic state close to the plateau of the Frank–Starling cardiac curve, fluid shifts induced by LC may not affect SV and CO [5]. During 60 mmHg LC, IAP increased by approximately 5.8 mmHg, and CO increased by 6.6%. Because there were no significant changes in HR during LC, the increase in CO was attributed to an increase in SV. Similarly, in one study conducted in pigs, an acute increase of 7.5 mmHg in IAP resulted in a 4.8% increase in CO, whereas another study showed that an increase of 10 mmHg in IAP increased the global end-diastolic volume, a marker of cardiac preload, from 15.5 to 17.0 mL/kg [6, 20].

In the present study, CO was measured using TTE. However, TTE is operator-dependent, potentially leading to inconsistent results, and compared to the standard thermodilution method, TTE may underestimate CO [21, 22]. Nevertheless, TTE allows real-time and continuous CO measurement and is recommended as the first evaluation in patients with circulatory failure due to its non-invasive nature [23]. CO is typically obtained in the apical five-chamber view by multiplying the VTI of the left ventricular outflow tract and the CSA of the aortic annulus [2, 4]. However, when the apical five-chamber view is not consistently maintained, there is a potential for underestimating the VTI [24]. Therefore, in the present study, CO was measured by multiplying the VTI of the mitral valve by the CSA of the mitral valve in the apical four-chamber view. A recent study in humans demonstrated that both measurement methods yielded similar results in cases without mitral valve regurgitation [24, 25].

Approximately 50% of IAP is transmitted to the thoracic cavity, and IAH can increase intra-thoracic pressure, thereby affecting respiratory parameters [20, 26]. In anesthetized, spontaneously breathing dogs, pneumoperitoneum with an IAP above 20 mmHg decreased VT and induced respiratory acidosis, whereas an IAP below 20 mmHg did not significantly affect RR, VT, MV, pH, PaO_2_, and PaCO_2_ [8, 27]. The mean IAP ranged from 1.7 to 6.3 mmHg at LC pressures of 10–44 mmHg, and LC did not significantly affect RR, VT, MV, pH, PaO_2_, and PaCO_2_, which is consistent with previous findings. During the 60 mmHg LC, despite the IAP remaining below 20 mmHg, the RR and pH significantly increased, while PaO_2_ and PaCO_2_ significantly decreased. The precise reason for the increased RR remains unclear. However, because there was no significant difference in the IAP between 44 and 60 mmHg, the possibility of increased RR due to elevated IAP seems minimal. Additionally, although not statistically significant, increased MV may have influenced the decreased PaCO_2_ and increased pH. When alveolar diffusion is normal, factors affecting PaO_2_ include the extent of tissue oxygen extraction, level of alveolar ventilation, intrapulmonary right-to-left shunting, and regions characterized by low ventilation-perfusion ratios [28]. However, in the present study, the possibility that IAP affected these factors was low. Furthermore, although statistically significant changes were observed in pH, PaO_2_, and PaCO_2_, the clinical impacts on oxygenation and ventilation were minimal.

Changes in blood pressure are related to changes in SV when the HR and SVR remain constant. A study in pediatric patients suggested that a significant increase in MAP with LC could predict an increase in SV [3, 4]. In hypovolemic dogs, the LC did not affect the HR and SVR; however, the MAP and SV increased significantly [5]. Similarly, in the present study, LC did not affect HR and SVR. However, studies on passive leg raising, which shares a similar mechanism, have suggested that monitoring blood pressure alone is not sufficient to assess temporary changes in SV and recommend measuring SV [29]. In addition, especially when IAP is increased, traditional pressure-based preload parameters such as CVP and pulmonary artery occlusion pressure do not reflect changes in preload, making the monitoring of volume-based parameters more appropriate [30]. In the present study, while LC significantly increased MAP at 44 and 60 mmHg, a significant increase in SV was observed only at 60 mmHg. This suggests that measuring SV may be more appropriate for evaluating the effects of LC.

This study has several limitations. First, due to ethical considerations, only a small number of animals were used. Second, all results were obtained from healthy, spontaneously breathing dogs under anesthesia, limiting the extrapolation of this study’s findings to various clinical scenarios. As such, further research is needed in various contexts including different breeds, conscious patients, critically ill patients, patients with abdominal masses, mechanically ventilated patients, and patients in different body positions. Third, CO was measured using TTE rather than thermodilution, which can be operator-dependent; however, to minimize bias, VTI measurements were conducted by the same operator. Fourth, the study was not conducted in a blinded and randomized sequence, which may have influenced the recording of hemodynamic and respiratory parameters.

In conclusion, LC ranging from 10 to 60 mmHg in healthy dogs under anesthesia with spontaneous breathing significantly increased IAP, but did not induce IAH. A LC of 22 mmHg resulted in a statistically significant increase of 1.7 mmHg in IAP, yet did not significantly affect CO and respiratory parameters. The findings of this study suggest that applying LC with commonly used pressure may pose a low risk of inducing IAH and associated complications. Further research is needed to use LC in various clinical settings.
